# EMOTION AND AGING: CUTTING-EDGE DIRECTIONS

**DOI:** 10.1093/geroni/igae098.1478

**Published:** 2024-12-31

**Authors:** Joseph Mikels, Laura Carstensen

**Affiliations:** Chair; Co-Chair

## Abstract

Over the past few decades, the field has garnered a deep and broad understanding of age differences in emotional experience; healthy older adults generally experience a balance of more positive to negative emotions relative to their younger counterparts. But many questions remain, and novel understandings of this general pattern are emerging. This symposium will highlight the latest cutting-edge directions in the examination of emotional functioning in later life. Mikels will present data demonstrating that older adults experience more “love” (i.e., positivity resonance) relative to younger adults. Haase and colleagues will explain how emotional acceptance is an important way by which older adults achieve greater well-being relative to other emotion regulation strategies. English and Springstein will further consider how the flexible use of different emotion regulation strategies contribute to higher well-being across the adult lifespan. In yet another approach to understanding high levels of well-being in older adults, Stephens will show that savoring is a critical component of increased positive affect in later life. Underscoring the social underpinnings of well-being, Brown and colleagues will explain the critical role of understanding other people’s emotions accurately in building and maintaining social connections, especially for older women. This series of presentations will illuminate the myriad current directions that are under investigation in the study of emotion and aging, providing attendees with a rich understanding of the latest findings.

